# Perceived Organizational Support Impacts on the Associations of Work-Family Conflict or Family-Work Conflict with Depressive Symptoms among Chinese Doctors

**DOI:** 10.3390/ijerph13030326

**Published:** 2016-03-16

**Authors:** Junhui Hao, Jiana Wang, Li Liu, Wei Wu, Hui Wu

**Affiliations:** 1 Department of Social Medicine, School of Public Health, China Medical University, No. 77 Puhe Road, Shenyang North New Area, Shenyang 110013, China; blink007@sina.cn (J.H.); jiana0818@163.com (J.W.); liul@mail.cmu.edu.cn (L.L.); 2 AstraZeneca China Management Academy, 28th Floor, 2807 Room, China Resources Building, No. 286, Young Street, Heping District, Shenyang 110014, China; wuweidavid888@163.com

**Keywords:** perceived organizational support, work-family conflict, family-work conflict, depressive symptoms, doctors

## Abstract

As a common mental disorder, depressive symptoms had been studied extensively all over the world. However, positive resources for combating depressive symptoms among Chinese doctors were rarely studied. Our study aimed to investigate the relationships between work-family conflict (WFC) and family-work conflict (FWC) with depressive symptoms among Chinese doctors. Meanwhile, the role of perceived organizational support (POS) in this association was explored at an organizational level. The investigation was conducted between March and April 2014. Questionnaires that measured WFC, FWC, depressive symptoms and POS were distributed to 1200 doctors in Shenyang, China. The final study subjects were 931 doctors (effective response rate: 77.6%). In all analyses, male and female doctors were analyzed separately because of possible gender differences. Hierarchical linear regression analyses were used to examine the moderating role of POS. Baron and Kenny’s technique and asymptotic and resampling strategies were used to explore the mediating role of POS on the associations of WFC or FWC with depressive symptoms. WFC and FWC had positive relations with depressive symptoms among doctors. POS played a partial mediating role on the correlation of FWC with depressive symptoms among male doctors, and POS played a partial mediating role on the correlation of WFC with depressive symptoms among female doctors. POS had a positive moderating effect on the relationship between WFC and depressive symptoms among doctors. WFC and FWC could aggravate doctors’ depressive symptoms, and POS, as an organizational resource, could fight against doctors’ depressive symptoms. When POS functioned as a mediator, FWC had a negative effect on POS, which could increase male doctors’ depressive symptoms, and WFC had a negative effect on POS, which could increase female doctors’ depressive symptoms. In the meantime, POS, as a moderator, could enhance the effects of WFC on depressive symptoms.

## 1. Introduction

As a common mental disorder, depressive symptoms had been studied extensively all over the world. People from every walk of life can be affected by depressive symptoms, such as college teachers, nurses and doctors [1,2,3,4]. Depressive symptoms not only threaten people’s health, but also affect the development of society [5]. As the major workforce in hospitals, doctors need to do heavy physical and psychological work, which may lead to depressive symptoms. Meanwhile, in China, the proportion of doctors to the general population is 1:735, which is lower than that in many Western developed countries (1:280–1:640) [5,6]. The enormous gap between doctors and the general population may become one of the factors causing Chinese doctors’ depressive symptoms. The prevalence of depressive symptoms among physicians ranged from 10% to 15% in the U.S., Britain, Norway and Japan. However, a study showed that the prevalence of depressive symptoms among physicians reached up to 65.3% in China [1]. Continuous and serious depressive symptoms may affect the professional ability of doctors and the quality of their work, which in turn may harm patients’ health and even life safety. Thus, exploring negative and positive factors of doctors’ depressive symptoms can not only help them find ways to improve their mental health, but also help hospitals provide a higher quality of health care services in China.

Work-family conflict is a two-way conflict that consists of work-family conflict (WFC) and family-work conflict (FWC) [7,8,9]. WFC is “a form of inter role conflict in which behavior, time devoted to and stress caused by the job interfere with family-related responsibilities,” and FWC is “a form of inter role conflict in which behavior, time devoted to and stress caused by the family interfere with work-related responsibilities”. Doctors often face challenging tasks in their working environment, requiring a great deal of time and energy, and they take responsibility for family at the same time. Thus, they are likely to experience a higher level of work-family conflict [10,11,12]. Previous studies showed that WFC and FWC had positive relations with depressive symptoms [13,14], and WFC had negative effects on psychological health of medical staff [15,16]. In China, a previous study showed that WFC was positively related to depressive symptoms among nurses [17]. Gender differences were revealed in the studies examining the associations of WFC and FWC with depressive symptoms. For example, a previous study demonstrated that WFC affected psychological capital for men, while FWC had an effect on psychological capital for women [18]. In China, few studies studied the relations of WFC and FWC with depressive symptoms between different genders simultaneously. Therefore, we aimed to examine the correlations of WFC and FWC with depressive symptoms among male and female Chinese doctors, separately. Besides determining the risk factors of depressive symptoms, more attention should be focused on exploring the positive resources to combat depressive symptoms.

Perceived organizational support (POS) is defined as “beliefs concerning the extent to which the organization values their contribution and cares about their well-being” [19]. POS is regarded as a highly effective organizational-based resource that causes positive work attitudes and outcomes, and has positive effects on people’s mental health [20,21]. It has been identified as a positive resource that can relieve depressive symptoms [22,23]. Meanwhile, some studies also showed that POS had a negative relation with WFC and FWC [23,24]. In addition, POS was identified as a mediator that could affect job satisfaction, turnover intentions, and depressive tendency [23,25,26]. 

For instance, a previous study found that POS mediated the relationship between effort-reward imbalance and depressive tendency among prison policemen [23]. POS was also identified as a moderator that affected the relationship between emotional labor and job-related outcomes [27]. Other studies showed that POS moderated the relationship between work-family conflict and job performance [24,28]. However, to date, there was no study that explored the indirect effect of POS on the associations of WFC and FWC with depressive symptoms. In this study, we hypothesized that POS had a mediating or moderating role in the associations of WFC or FWC with depressive symptoms among Chinese doctors. The study had three purposes: first, the associations of WFC and FWC with depressive symptoms would be examined in Chinese female and male doctors, separately. Second, we would test whether POS moderated the effect of WFC or FWC on depressive symptoms. Third, we would test whether POS mediated the effect of WFC or FWC on depressive symptoms.

## 2. Methods

### 2.1. Ethics Statement

The study procedures were in line with the ethical standards of the Committee on Human Experimentation of China Medical University, and the Committee on Human Experimentation of China Medical University approved this study (CMU62083011). The written informed consent was obtained from each participant who voluntarily participated in the study. We protected the privacy and anonymity of individuals involved in our research.

### 2.2. Study Design and Data Collection

A prevalence survey was conducted in Shenyang, Liaoning Province, in the northeastern region of China. Given the geographic characteristics, the whole city was divided five regions (eastern, western, southern, northern, and central). One large hospital (>500 beds) was selected randomly in each sampled region. Half of the doctors from each selected hospital were chosen randomly. Self-administered questionnaires were distributed to 1200 recruited doctors in 2014, and 986 questionnaires were returned. After excluding invalid questionnaires, 931 doctors (423 males and 508 females, effective response rate: 77.6%) became the final subject in the current study.

### 2.3. Measurement of Depressive Symptoms

The Center for Epidemiologic Studies Depression Scale (CES-D) was chosen to evaluate the depressive symptoms of doctors [29,30]. The Chinese version of the questionnaire was widely used in Chinese populations because its good reliability and validity [31,32]. Every item consists of four viable options: (0) never; (1) sometimes; (2) frequently; and (3) always, and a total score of 60. The standard CES-D Scale defined the depressive symptoms when a total CES-D score ≥16. In this study, for males, Cronbach’s alpha for the CES-D was 0.902. For female doctors, Cronbach’s alpha was 0.908. Meanwhile, in this study, for males, a satisfactory goodness-of-fit of CES-D was confirmed through using the confirmatory factory analysis (RMSEA = 0.062, CFI = 0.944, GFI = 0.906). For females, a satisfactory goodness-of-fit of CES-D was confirmed through using the confirmatory factory analysis (RMSEA = 0.069, CFI = 0.931, GFI = 0.901).

### 2.4. Measurement of WFC and FWC

Work-family conflict included two scales: the WFC scale and FWC scale [7]. The WFC scale measures the content to which work has an influence in family, while the FWC scale measures the content to which family has an influence in work. The total scale includes 18 items, and each dimension was measured by nine items. Each of the items is scored on a Likert scale in which 1 means never and 5 means always. Responses for both WFC scale and FWC scale were summed, and get the results to take an average score for WFC and FWC, respectively. Higher values forecast higher levels of WFC and FWC. Good reliability and validity of the Chinese version of the work-family scale was showed in previous studies [17,18,33]. In this study, for males, the Cronbach’s alpha was 0.903 (WFC) and 0.904 (FWC). For females, the Cronbach’s alpha was 0.882 (WFC) and 0.907 (FWC). Meanwhile, in this study, for males, a satisfactory goodness-of-fit of WFC and FWC scales was confirmed through using the confirmatory factory analysis (RMSEA = 0.038, CFI = 0.995, GFI = 0.983; RMSEA = 0.067, CFI = 0.983, GFI = 0.969). For females, a satisfactory goodness-of-fit of WFC and FWC scales was confirmed through using the confirmatory factory analysis (RMSEA = 0.059, CFI = 0.987, GFI = 0.974; RMSEA = 0.076, CFI = 0.980, GFI = 0.965).

### 2.5. Measurement of POS

POS was measured by Survey of Perceived Organizational Support (SPOS) [19,34]. The scale includes 9-item, which indicates the organization’s valuation and well-being of employees. Each of the items is scored on a Likert scale in which 1 means strongly disagree and 7 means strongly agree. In this study, Responses for POS are the average score of the 9 items. Higher values evaluated higher levels of POS. Good reliability and validity of the Chinese the 9-item version of POS scale was showed in previous studies [22,35]. For males, the Cronbach’s alpha for the POS scale was 0.891, and for females, the Cronbach’s alpha for the POS scale was 0.888. Meanwhile, in this study, for males, a satisfactory goodness-of-fit of POS was confirmed through using the confirmatory factory analysis (RMSEA = 0.072, CFI = 0.985, GFI = 0.967). For females, a satisfactory goodness-of-fit of POS was confirmed through using the confirmatory factory analysis (RMSEA = 0.064, CFI = 0.987, GFI = 0.971).

### 2.6. Demographic Characteristics

Including four demographic characteristics were marital status, gender, age, education, having children, and weekly work time, respectively in this study. Marital status was categorized as single and married/cohabitation. Education was categorized as junior college, college and graduate or higher. Age was categorized as ≤30, 31–39, and ≥40. Having children was categorized as yes and no. Weekly work time was categorized as ≤40 and >40.

### 2.7. Statistical Analysis

In this study, SPSS 17.0 (SPSS China Corp., Shanghai, China) was used to analyze data. Pearson’s Chi-square (χ^2^) tests were used to compare differences in demographic characteristics between male and female doctors. *T*-test and one-way ANOVA analyses were used to examine the difference of study variables among age groups, education groups, and marital status groups. All statistical tests were two-sided (α = 0.05).

The whole successive variables including the independent variables and the mediating variables were centralized to examine the moderating role ahead of regression analyses [36]. We used Pearson’s correlation to examine relations among successive variables. Hierarchical linear regression analyses were used to explore the moderating effect of POS in the associations of WFC or FWC with depressive symptoms. In step 1 of the analyses, age used as the covariate. In step 2, WFC/FWC and POS were added. In step 3, WFC × POS or FWC × POS was added.

**Figure 1:**
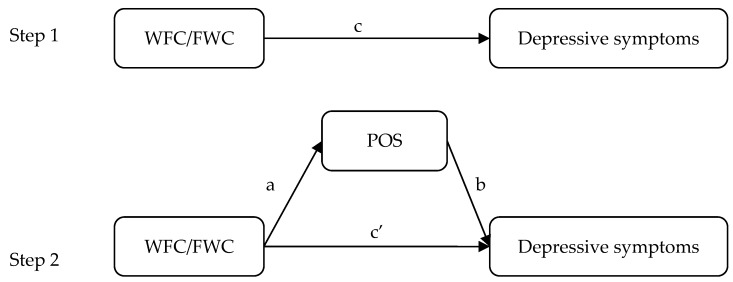
Theoretical model of the mediating effect of POS on the associations of WFC, FWC and depressive symptoms. (**a**) the associations of WFC, FWC and POS; (**b**) the correlation of POS with depressive symptoms after controlling for the predictor variable; (**c**) the correlations among WFC, FWC with depressive symptoms; (**c’**) the correlations among WFC, FWC and depressive symptoms after adding POS.

Meanwhile, POS may be a potential mediator of the correlations among WFC, FWC and depressive symptoms, and Baron and Kenny’s technique and asymptotic and resampling strategies were used to test its effect [37,38]. WFC or FWC was regarded as independent variables, depressive symptoms were regarded as the dependent variable, and POS was a mediator (as shown in Figure 1). In the first step, the purpose is to certify the correlations among WFC, FWC and depressive symptoms (the c path), and the second step is to test the mediation of POS (the a × b path). If the c’ path coefficient in the second step was smaller than the c path coefficient in the first step, or was not significant, the mediation of POS may exist. Five thousand bootstrap samples were used to access the presented study. A bias-corrected and accelerated 95% confidence interval (BCa 95% CI) was determined for each a × b product, and a BCa 95% CI excluding 0 indicated significant mediation. In all analyses, male doctors and female doctors need to be analyzed separately because of possible gender differences.

## 3. Result

### 3.1. Participant Characteristics

As shown in Table 1 and Table 2, in all demographic groups, there were significant differences in age, marital status and having children between male and female doctors, but there were no differences for depressive symptoms whatever the subjects are males or females.

### 3.2. Correlations between Continues Variables

Correlations among the continues variables were showed in Table 3. Both WFC and FWC had significant and positive correlations with depressive symptoms for males and females. For males, FWC had a negative relation with POS, but WFC was no significantly with POS. For females, both WFC and FWC had negative correlations with POS. Therefore, we will test the mediating effect of POS on the relation of WFC with depressive symptoms among female doctors, and examine the mediating effect of POS on the relation of FWC with depressive symptoms among male doctors and female doctors, separately. 

### 3.3. The Interaction of WFC/FWC and Gender for Doctors

As shown in Table 4, for doctors, in step 2, WFC has no significant relation with depressive symptom, while adding the interaction of WFC and gender. The interaction of WFC/FWC and gender have no significant relations with depressive symptoms.

### 3.4. The Moderation of POS on the Associations of WFC or FWC with Depressive Symptoms among Male Doctors

As shown in Table 5, for male doctors, after adding the covariate, WFC was positively related with depressive symptoms (β = 0.219, *p* < 0.01), while POS had a negative relation with depressive symptoms (β = −0.377, *p* < 0.01). The interaction of WFC and POS had a positive relation with depressive symptoms (β = 0.094, *p* < 0.05). Therefore, POS moderated on the relationship between WFC and depressive symptoms (β = 0.094, *p* < 0.05). Meanwhile, for male doctors, after adding the covariate, FWC was positively related with depressive symptoms (β = 0.405, *p* < 0.01), while POS had a negative relation with depressive symptoms (β = −0.319, *p* < 0.01). The interaction of FWC and POS had no significant relation with depressive symptoms (β = 0.064, *p* > 0.05). Thus, POS had no moderating effect on the relationship between FWC and depressive symptoms (β = 0.064, *p* > 0.05).

### 3.5. The Moderation of POS on the assocIations of WFC or FWC with Depressive Symptoms among Female Doctors

As shown in Table 6, for female doctors, after adding the covariate, WFC was positively related with depressive symptoms (β = 0.299, *p* < 0.01), while POS had a negative relation with depressive symptoms (β = −0.235, *p* < 0.01). The interaction of WFC and POS had a positive relation with depressive symptoms (β = 0.114, *p* < 0.01). Therefore, POS moderated on the relationship between WFC and depressive symptoms (β = 0.114, *p* < 0.01). Meanwhile, for female doctors, after adding the covariate, FWC was positively related with depressive symptoms (β = 0.476, *p* < 0.01), while POS had a negative relation with depressive symptoms (β = −0.244, *p* < 0.01). The interaction of FWC and POS had no significant relation with depressive symptoms (β = 0.027, *p* > 0.05). Thus, POS had no moderating effect on the association between FWC and depressive symptoms (β = 0.027, *p* > 0.05).

### 3.6. The Mediation of POS on the Association between WFC and Depressive Symptoms among Female Doctors

As shown in Table 7, in step 2, WFC was positively related with depressive symptoms (β = 0.342, *p* < 0.01). In step 3, POS was negatively related with depressive symptoms (β = −0.235, *p* < 0.01). The effect of WFC on depressive symptoms in step 3 was reduced compared with that in step 2, when POS were added (β = 0.299, *p* < 0.01). Thus, POS may plays a partially mediating role in the relationship between WFC and depressive symptoms.

As shown in Table 8, for female doctors, the relationship between WFC and depressive symptoms (the c path) was tested. WFC was positively correlated with depressive symptoms. The mediating effect of POS on the relation of WFC with depressive symptoms was then tested.

WFC had a negative relation with POS (the a path). Then, POS was negatively correlated with depressive symptoms after controlling for WFC (the b path). Meanwhile, the direct pathways of WFC with depressive symptoms (the c’ path) was significantly while POS was added in the model, and a BCa 95% CI did not include 0. Thus, POS had a partially mediating effect on the relationship between WFC and depressive symptoms for female doctors.

We calculated the proportion of the total effect of the independent variable on the outcome variable (c) that was mediated by POS using the formula (a × b)/c. The proportion of POS mediating effect was 13.0% for WFC.

### 3.7. The Mediation of POS on the Association between FWC and Depressive Symptoms Either Male or Female Doctors

As shown in Table 9, for male doctors, in step 2, FWC was positively related with depressive symptoms (β = 0.464, *p* < 0.01). In Step 3, POS was negatively related with depressive symptoms (β = −0.319, *p* < 0.01). The effect of FWC on depressive symptoms in step 3 was reduced compared with that in step 2, when POS were added (β = 0.405, *p* < 0.01). Meanwhile, for female doctors, in step 2, FWC was positively related with depressive symptoms (β = 0.498, *p* < 0.01). In step 3, POS was negatively related with depressive symptoms (β = −0.244, *p* < 0.01). The effect of FWC on depressive symptoms in step 3 was reduced compared with that in step 2, when POS were added (β = 0.476, *p* < 0.01). Thus, POS may plays a partially mediating role in the relationship between FWC and depressive symptoms among doctors.

As shown in Table 10, for male and female doctors, the relationship between FWC and depressive symptoms (the c path) was tested. FWC was positively correlated with depressive symptoms. The mediating effect of POS on the relation of FWC with depressive symptoms was then tested.

FWC had a significant correlation with POS (the a path). Then, POS was negatively correlated with depressive symptoms after controlling for FWC (the b path), and the effect of FWC on depressive symptoms (the c’ path) was decreased. However, for male doctors, a BCa 95% CI did not include 0, and for female doctors, a BCa 95% CI did include 0. Thus, POS had a partially mediating effect on the relationship between FWC and depressive symptoms for male doctors, and POS had no mediating effect on the relationship between FWC and depressive symptoms for female doctors.

We calculated the proportion of the total effect of the independent variable on the outcome variable (c) that was mediated by POS using the formula (a × b)/c. For males doctors, the proportion of POS mediating effect was 12.5% for FWC.

## 4. Discussion

This study explored the relations of WFC and FWC with depressive symptoms and the mediating and moderating effects of POS in the relations among Chinese doctors. The large number of studied doctors from hospitals in Shenyang, China, had similar working environments and incomes based on different positions. Moreover, the sample had an effective response rate (77.6%), which could be considered a good representation of our study population.

In accordance with several studies [10,14,39,40,41], WFC and FWC were found to have a positive correlation with depressive symptoms among doctors in this study. The explanation is that high levels of work-family conflict may lead to negative work-related factors and poor working conditions, and all these negative work-related factors can cause psychological depression. In other words, people who have more work-family conflicts devote more time and energy to working at the expense of interfering with family-related responsibilities, and they usually do not have enough time to relieve work pressure. As a result, continuous pressure exhausts their body and mind [42,43,44,45]. For Chinese doctors, they take more responsibilities because the ratio of doctors to the general population is much smaller than many other countries. Meanwhile, although both doctors and nurses are medical staffs, doctors often suffer higher level of stress because their work is more closely correlated with patients’ health. They often devote themselves to their patients rather than families. These factors may increase the levels of WFC and FWC, which in turn cause depressive symptoms among doctors [12,46,47]. Therefore, hospital administrators should pay more attention to the risk factors of WFC and FWC. They should provide a satisfying working environment and appropriate working hours so that doctors can spend more time with their families, which can reduce the levels of WFC. In order to get support and receive understanding from family, doctors should communicate with family members, and tell them the importance of their present work. By these means, the levels of FWC may be reduced. 

POS, as a positive resource, can reduce the levels of negative mental factors, such as workplace stressors, job-related burnout and depression [20]. In this study, POS had a negative relation with depressive symptoms among Chinese doctors, which showed that people could improve POS to reduce depressive symptoms. In addition to its direct effect, our study is the first one to explore the mediating and moderating effect of POS on the relations of WFC and FWC with depressive symptoms.

In this study, the mediation effects of POS showed a gender difference. For male doctors, our study revealed that POS had a partial mediating effect on the relationship between FWC and depressive symptoms, and POS had no mediating effect on the relationship between WFC and depressive symptoms. That means that FWC had a negative effect on POS, which could increase male doctors’ depressive symptoms. However, for female doctors, POS had a partial mediating effect on the relationship between WFC and depressive symptoms, and POS had no mediating effect on the relationship between FWC and depressive symptoms. This result demonstrated that female doctors who felt more WFC could be likely to experience lower levels of POS, which might enhance depressive symptom. The reason is that different forms of work division may lead to different levels of stress or conflicts. Male doctors often regard work as their primary responsibility, and compared with their female counterparts, they usually spent more time and energy on their work rather than on their families [15,17,32]. That means male doctors could feel more POS than females in hospitals. Thus, when male doctors feel more WFC, POS does work in terms of deceasing depressive symptoms. On the contrary, POS does not work to reduce depressive symptoms among male doctors when they feel more FWC. Meanwhile, most female doctors spent more time looking after their families [12,46]. It means that females probably feel lower level of POS when they feel more FWC, contributing to female doctors’ depressive symptoms. Therefore, organizations should make an effort to ensure adequate support for their employees in order to develop positive beliefs in them. Hospital leaders can deliver the information to employees that the organization genuinely and sincerely concerns about them, and can also increase their rewards. Moreover, frequent communications are significant between leaders and employees, as it can help organization to know their employees’ confusions, and offer its help [48]. 

In addition, our study showed that POS had a positive moderating role on the relationship between WFC and depressive symptoms among doctors. When doctors felt a high level of POS, depressive symptoms increased with the increase of WFC. However, when doctors felt a low level of POS, depressive symptoms did not increase obviously with the increase of WFC. Therefore, to effectively reduce depressive symptoms, hospital managers can both improve the level of POS and reduce the level of WFC through some approaches.

## 5. Conclusions

The present study showed that WFC and FWC could increase doctors’ depressive symptoms, and POS, as a positive resource, could fight against doctors’ depressive symptoms. In the meantime, POS, as a moderator, could enhance the effects of WFC on depressive symptoms. When POS functioned as a mediator, FWC had a negative effect on POS, which could increase male doctors’ depressive symptoms, and WFC had a negative effect on POS, which could increase female doctors’ depressive symptoms. Some limitations of this should be acknowledged. First, the present study is a cross-sectional design. Because WFC, FWC, POS and depressive symptoms were measured simultaneously, it is impossible to draw causal conclusions. Second, all the subjects were from large general hospitals, and doctors from small-scale hospitals or community health centers were not covered. In future research, doctors of these institutions should be considered. Third, this study just considered the relation of WFC and FWC with depressive symptoms and controlled some basic social demographics. Other information, such as the number of children, spouse occupation, was not collected and might have some effects on the results. The possible confounders and some risk factors need to be studied in future.

## Figures and Tables

**Table 1:** Demographic characteristics and distributions of doctors.

Variable	Males(423) N (%)	Females(508) N (%)	*p*-Value
Age (years)			<0.01
≤30	104 (24.6%)	191 (37.6%)	
30–40	170 (40.2%)	161 (31.7%)	
≥40	149 (35.2%)	156 (30.7%)	
Marital status			<0.01
Single/widowed/divorced	85 (20.1%)	154 (30.3%)	
Married/cohabiting	338 (79.9%)	354 (69.7%)	
Education			0.316
Junior college or lower	45 (10.6%)	55 (10.8%)	
College	256 (60.5%)	284 (55.9%)	
Graduate or higher	122 (28.8%)	169 (33.3%)	
Having children			<0.01
Yes	79 (18.7%)	144 (28.3%)	
No	344 (81.3%)	364 (71.7%)	
Weekly work time			0.111
≤40	113 (26.7%)	160 (31.5%)	
>40	310 (73.3%)	348 (68.5%)	

**Table 2:** Demographic characteristics and distributions of depressive symptoms.

Variable	Males		Females	
	N (%)	Depressive Symptoms	N (%)	Depressive Symptoms
		Mean (SD)		Mean (SD)
Total	423 (45.4%)	19.54 (10.34)	508 (54.6%)	19.39 (10.22)
Age (years)				
≤30	104 (24.6%)	18.70 (11.02)	191 (37.6%)	18.94 (10.41)
30–40	170 (40.2%)	20.20 (10.47)	161 (31.7%)	20.42 (10.46)
≥40	149 (35.2%)	19.38 (9.70)	156 (30.7%)	18.89 (9.71)
Marital status				
Single/widowed/divorced	85 (20.1%)	19.74 (11.88)	154 (30.3%)	19.20 (9.56)
Married/cohabiting	338 (79.9%)	19.49 (9.93)	354 (69.7%)	19.48 (10.501)
Education				
Junior college or lower	45 (10.6%)	19.40 (10.59)	55 (10.8%)	19.63 (10.85)
College	256 (60.5%)	20.09 (10.61)	284 (55.9%)	19.51 (9.38)
Graduate or higher	122 (28.8%)	18.43 (9.63)	169 (33.3%)	19.13 (11.35)
Having Children				
Yes	79 (18.7%)	19.50 (11.55)	144 (28.3%)	19.16 (9.66)
No	344 (81.3%)	19.55 (10.06)	364 (71.7%)	19.49 (10.44)
Weekly work time				
≤40	113 (26.7%)	19.28 (10.62)	160 (31.5%)	19.28 (10.03)
>40	310 (73.3%)	19.63 (10.25)	348 (68.5%)	19.44 (10.32)

SD: standard deviations.

**Table 3:** Correlations of continues variables for doctors.

Variables	Males					Females
	1	2	3	4	5	6
1. Age (years)	-	−0.043	0.003	−0. 022	0.040	−0.14 **
2. Depressive symptoms	−0.001	-	0. 324 **	0.494 **	−0.291 **	−0.019
3. WFC	0.018	0.238 **	-	0.441 **	−0.173 **	0.202 **
4. FWC	−0.036	0.463 **	0.541 **	-	−0.090 *	0.016
5. POS	0.057	−0.390 **	−0.077	−0.185 **	-	0.046
6. Weekly work time	−0.172 **	−0.018	0.153 **	−0.051	−0.028	-

* *p* < 0.05, ** *p* < 0.01 (two-tailed). Results for male doctors are below the diagonal and for female doctors are above the diagonal.

**Table 4:** The interaction of WFC/FWC and gender for doctors.

Variable	Depressive Symptoms				Variable	Depressive Symptoms			
	step1 (β)	*p*-value	step2 (β)	*p*-value		step1 (β)	*p*-value	step2 (β)	*p*-value
Age (years)	−0.048	0.219	−0.046	0.230	Age (years)	−0.035	0.323	−0.036	0.316
Weekly work time	−0.078 *	0.017	−0.080 *	0.014	Weekly work time	−0.015	0.610	−0.016	0.595
Marital status	−0.044	0.679	−0.039	0.714	Marital status	−0.120	0.219	−0.123	0.207
Having children	0.056	0.609	0.049	0.653	Having children	0.151	0.131	0.155	0.123
Gender	−0.021	0.504	−0.273	0.048	Gender	0.013	0.657	−0.049	0.625
WFC	0.295 **	0.000	0.119	0.228	FWC	0.481 **	0.000	0.423 **	0.000
WFC × Gender	-	-	0.317	0.060	FWC × Gender	-	-	0.085	0.518
R2	0.086	-	0.090	-	R2	0.223	-	0.223	-
ΔR2	0.086 **	-	0.090	-	ΔR2	0.223 ^**^	-	0.000	-

* *p* < 0.05, ** *p* < 0.01 (two-tailed).

**Table 5:** The regression analysis for the interaction of WFC, FWC and POS among male doctors.

Variable	Depressive Symptoms			Variable	Depressive Symptoms		
	step1 (β)	step2 (β)	step3 (β)		step1 (β)	step2 (β)	step3 (β)
Age (years)	−0.009	0.013	0.01	Age (years)	−0.009	0.04	0.039
Weekly work time	−0.019	−0.058	−0.058	Weekly work time	−0.019	0.003	0.009
Marital status	−0.135	−0.176	−0.175	Marital status	−0.135	−0.132	−0.129
Having children	0.136	0.153	0.15	Having children	0.136	0.108	0.103
WFC	-	0.219 **	0.242 **	FWC	-	0.405 **	0.412 **
POS	-	−0.377 **	−0.370 **	POS	-	−0.319 **	−0.308 **
WFC×POS	-	-	0.094 *	FWC×POS	-	-	0.064
R2	0.002	0.202	0.21	R2	0.002	0.313	0.317
ΔR2	0.002	0.200 **	0.008 *	ΔR2	0.002	0.311 **	0.004

* *p* < 0.05, ** *p* < 0.01 (two-tailed).

**Table 6:** The regression analysis for the interaction of WFC, FWC and POS among female doctors.

Variable	Depressive Symptoms			Variable	Depressive Symptoms		
	step1 (β)	step2 (β)	step3 (β)		step1 (β)	step2 (β)	step3 (β)
Age (years)	−0.085	−0.043	−0.039	Age (years)	−0.085	−0.053	−0.051
Weekly work time	−0.027	−0.075	−0.067	Weekly work time	−0.027	−0.017	−0.017
Marital status	−0.025	0.008	0.011	Marital status	−0.025	−0.157	−0.160
Having children	0.088	−0.010	−0.014	Having children	0.088	0.193	0.193
WFC	-	0.299 **	0.291 **	FWC	-	0.476 **	0.473 **
POS	-	−0.235 **	−0.232 **	POS	-	−0.244 **	−0.238 **
WFC × POS	-	-	0.114 **	FWC × POS	-	-	0.027
R2	0.005	0.168	0.181	R2	0.005	0.310	0.311
ΔR2	0.005	0.163 **	0.013 **	ΔR2	0.005	0.305 **	0.001

** *p* < 0.01 (two-tailed).

**Table 7:** Hierarchical linear regression analysis results for WFC, POS and depressive symptoms among female doctors.

Variable	Depressive Symptoms		
	step1(*β*)	step2(*β*)	step3(*β*)
Age	−0.085	−0.071	−0.043
Weekly work time	−0.027	−0.097 *	−0.075
Marital status	−0.025	0.029	0.008
Having children	0.088	−0.004	−0.010
WFC	-	0.342 **	0.299 **
POS	-	-	−0.235 **
R2	0.005	0.116	0.168
ΔR2	0.005	0.111 **	0.052 **

* *p* < 0.05, ** *p* < 0.01 (two-tailed).

**Table 8:** Regression analysis results, with WFC as the independent variable, and POS as a mediator.

Predictors	Path Coefficients				a × b (BCa 95% CI)
	c	a	b	c’	
Females	-	-	-	-	-
POS	0.324 **	‒0.173 **	‒0.241**	0.283 **	0.042 (0.017, 0.074)

** *p* < 0.01 (two-tailed).

**Table 9:** Hierarchical linear regression analysis results for FWC, POS and depressive symptoms.

Variable	Depressive Symptoms					
	Males			Females		
	step1(β)	step2(β)	step3(β)	step1(β)	step2(β)	step3(β)
Age	−0.009	0.020	0.040	−0.085	−0.084	−0.053
Weekly work time	−0.019	0.010	0.003	−0.027	−0.032	−0.017
Marital status	−0.135	−0.093	−0.132	−0.025	−0.147	−0.157
Having children	0.136	0.082	0.108	0.088	0.217	0.193
FWC	-	0.464 **	0.405 **	-	0.498 **	0.476 **
POS	-	-	−0.319 **	-	-	−0.244 **
R2	0.002	0.216	0.313	0.005	0.252	0.302
ΔR2	0.002	0.214 **	0.097 **	0.005	0.247 **	0.058 **

** *p* < 0.01 (two-tailed).

**Table 10:** Regression analysis results, with FWC as the independent variable, and POS as a mediator.

Predictors	Path Coefficients				a × b (BCa 95% CI)
	c	a	b	c’	
Males	-	-	-	-	-
POS	0.464 **	‒0.183 **	‒0.317 **	0.406 **	0.058 (0.025, 0.101)
Females	-	-	-	-	-
POS	0.494 **	‒0.089 *	‒0.248 **	0.472 **	0.022 (‒0.002, 0.052)

* *p* < 0.05, ** *p* < 0.01 (two-tailed).
